# Polyethylene Glycol (PEG) Linked to Near Infrared (NIR) Dyes Conjugated to Chimeric Anti-Carcinoembryonic Antigen (CEA) Antibody Enhances Imaging of Liver Metastases in a Nude-Mouse Model of Human Colon Cancer

**DOI:** 10.1371/journal.pone.0097965

**Published:** 2014-05-23

**Authors:** Ali A. Maawy, Yukihiko Hiroshima, Yong Zhang, George A. Luiken, Robert M. Hoffman, Michael Bouvet

**Affiliations:** 1 Department of Surgery, University of California San Diego, San Diego, California, United States of America; 2 AntiCancer, Inc., San Diego, California, United States of America; 3 Yokohama City University, Yokohama City, Japan; 4 OncoFluor, Inc., San Diego, California, United States of America; 5 VA San Diego Healthcare System, San Diego, California, United States of America; National Cancer Institute, United States of America

## Abstract

We report here that polyethylene glycol (PEG) linked to near infrared dyes conjugated to chimeric mouse-human anti-carcinoembryonic antigen (CEA) antibody greatly improves imaging of liver metastases in a nude mouse model of colon-cancer experimental metastases. PEGylated and non-PEGylated DyLight 650 and 750 dyes were conjugated to the chimeric anti-CEA antibody. The dyes were initially injected intravenously into nude mice without tumors. Tissue biodistribution was determined by tissue sonication and analyzing tissue dye concentration profiles over time. PEGylated dyes had significantly lower accumulation in the liver (p = 0.03 for the 650 dyes; p = 0.002 for the 750 dyes) compared to non-PEGylated dyes. In an experimental liver metastasis model of HT-29 colon cancer, PEGylated dyes conjugated to the anti-CEA antibody showed good labeling of metastatic tumors with high contrast between normal and malignant tissue which was not possible with the non-PEGylated dyes since there was so much non-specific accumulation in the liver. PEGylation of the DyLight 650 and 750 NIR dyes significantly altered tissue biodistribution, allowing brighter tissue labeling, decreased accumulation in normal organs, particularly the liver. This enabled high fidelity and high contrast imaging of liver metastases.

## Introduction

The use of fluorescence imaging has become widespread in mouse models of cancer and has begun to make an impact in the clinical care of patients [1], [2]. Fluorescently-tagged tissues of interest allow for easy detection and demarcation from other structures in both living and fixed tissues. In living tissue this allows for differential imaging of cells or molecules without disrupting other biological processes and also allows for real-time imaging [1].

Fluorescence-guided surgery (FGS), using a variety of techniques, has been evaluated in mouse models and humans and has been shown to facilitate evaluation of critical structures and improved resection of tumors [3]–[11]. In an effort to bring FGS to the clinic, we have developed FGS in orthotopic mouse models of pancreatic and colon cancer by labeling the tumor with tumor-specific monoclonal mouse antibodies conjugated to Alexa Fluor 488, a green fluorophore [12], [13] as well as labeling with a tumor-specific adenovirus [11]. In subsequent studies, minimally invasive fluorescence laparoscopy was used to successfully image orthotopic pancreatic tumors in mice labeled with fluorophore conjugated monoclonal mouse antibodies to CEA [14], [15].

The chimeric anti-CEA antibody used in the present study comprises a murine variable domain and a human constant domain. As non-human proteins, mouse antibodies tend to evoke an immune reaction if administered to humans. The chimerization process involves engineering the replacement of segments of the antibody molecule that distinguish it from a human antibody. The chimeric anti-CEA antibody has a human constant domain, thereby eliminating most of the potentially immunogenic portions of the antibody without altering its specificity for the intended target.

In recent studies in an orthotopic model of pancreatic cancer, we conjugated the chimeric anti-CEA antibody to variety of visible and NIR dyes to evaluate their ability to penetrate skin, peritoneum, and liver. We observed uptake of antibody-dye conjugate in the liver which hampered the ability to image liver metastases [16].

Polyethylene glycol (PEG) conjugation has been employed to decrease immunogenicity and improve pharmacokinetics of many drugs, peptides and compounds [17]. PEGylation is currently considered one of the most effective methods of prolonging the residence time of biologically active proteins in the bloodstream [18]–[20]. We hypothesized that by using a PEGylated NIR dye conjugated to a chimeric anti-CEA antibody, hepatic accumulation of the dye-antibody conjugate could be prevented resulting in a high signal to background ratio (SBR) and thus facilitating the imaging of hepatic metastases.

## Materials and Methods

### Cell Culture

The human colon cancer cell line HT-29 was obtained from the American Type Culture Collection (Manassas, VA) and was maintained in RPMI 1640 medium supplemented with 10% fetal bovine serum (FBS) and 2 mM glutamine from Gibco-BRL, Life Technologies, Inc. (Grand Island, NY). Cells were cultured at 37°C in a 5% CO_2_ incubator. The HT-29 cells were stably transfected with red fluorescent protein (RFP) or green fluorescent protein (GFP) as previously described [1], [10], [21].

### Antibody-Dye Conjugation

**Figure 1:**
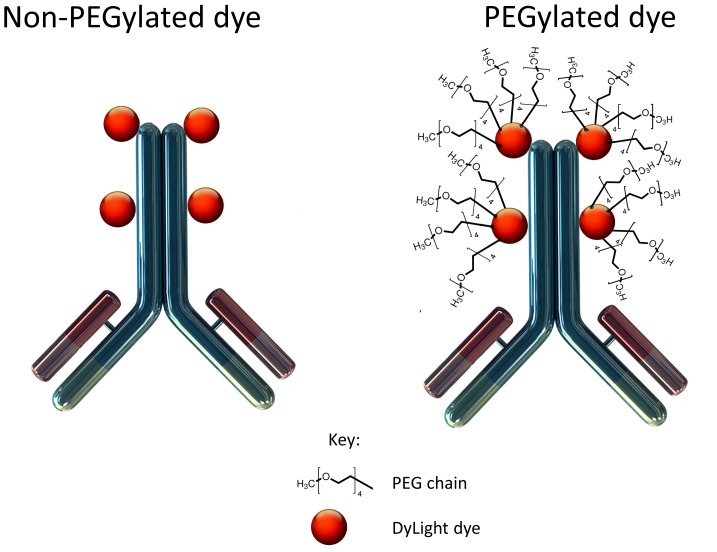
Schematic comparison of anti-CEA chimeric antibodies with PEGylated and non-PEGylated NIR dyes. Four mPEG200 chains are covalently linked to each dye molecule, 4 of which are covalently linked to the chimeric anti-CEA antibody by amide bonds. The mPEG chains alter tissue biodistribution, allowing brighter liver metastases labeling and decreased accumulation in normal organs, particularly the liver.

Chimeric anti-CEA antibody (Aragen Biosciences, Morgan Hill, CA) was conjugated to PEGylated and non-PEGylated DyLight dyes (Thermo Fisher Scientific, Rockford, IL) per manufacturer specifications, ensuring a minimum dye:protein ratio of at least 4∶1 (Figure 1). For each mole of antibody, there were 4 moles of dye. Protein:dye concentrations and ratios were confirmed using a NanoDrop Spectrophotometer (Thermo Fisher Scientific, Rockford, IL).

### Animal Care

Athymic *nu/nu* nude mice (AntiCancer, Inc., San Diego, CA) between 4 and 6 weeks of age were maintained in a barrier facility on high efficiency particulate air (HEPA)-filtered racks. The animals were fed with an autoclaved laboratory rodent diet (Teckland LM-485; Western Research Products, Orange, CA). All surgical procedures and imaging were performed with the animals anesthetized by intramuscular injection of 0.02 ml of a solution of 50% ketamine, 38% xylazine and 12% acepromazine maleate. When inhalational anesthesia was required, 2% isoflurane was delivered via a vaporizer. All animal studies were conducted with an AntiCancer Institutional Animal Care and Use Committee (IACUC)-protocol specifically approved for this study and in accordance with the principals and procedures outlined in the National Institute of Health Guide for the Care and Use of Animals under Assurance Number A3873-1. Animals received Buprenorphine 0.10 mg/kg ip immediately prior to surgery and once a day over the next 3 days to ameliorate suffering. CO_2_ inhalation was used for euthanasia. To ensure death following CO_2_ asphyxiation, cervical dislocation was performed.

### Tissue Biodistribution

2.5 nmol (94 µg) of dye conjugated to anti-CEA antibody in 100 µl PBS was injected into the tail vein of 60 nude mice. Three mice were then subsequently sacrificed at various time points: 5 minutes, 30 minutes, 1 hour, 3 hour, 6 hour and 24 hours. An incision was made from the sternum to the lower abdomen and the whole mouse opened to ensure complete visualization of the viscera. Imaging was performed using the OV-100 Small Animal Imaging System (Olympus, Tokyo, Japan).

From the same group of mice 5 mg of liver tissue was obtained. In each case 200 µl PBS was added to the tissue, which was subsequently homogenized by sonication with a probe Branson 450 sonifier (Branson Ultrasonics Corp., Danbury, CT) at 20 KHz until complete liquefaction.

The homogenized tissue was subsequently centrifuged at 5000 rpm for 3 minutes and the supernatant with dye collected. 100 µl of each sample was placed in a 96-well plate and diluted to 200 µl. The sample was placed in the plate reader and the absorbance from each sample collected. Using standard curves, the sample concentration of dye was determined, allowing for determination of dye concentration in the tissue in nmol/mg. Tissue concentrations of each dye were plotted over time.

### Liver metastasis formation and labeling

HT-29-RFP colon cancer cells (1×10^6^) were injected into the spleen of nude mice as previously described [22]. The cells trafficked in the liver parenchyma and formed liver metastases over a period of 4 weeks. After ensuring the presence of liver metastases by non-invasive whole body imaging, the mice were injected with anti-CEA antibody conjugated to either DyLight 650-PEG or DyLight 750-PEG, or conjugated to the non-PEGylated equivalent dyes. Two mice were used per group. Twenty-four hours later, the mice were sacrificed and a ventral vertical incision from the sternum to the lower abdomen made to facilitate complete visceral exposure for imaging.

To further determine the specificity of the conjugated antibodies, mice with HT-29-GFP liver metastases as described above were treated as follows: Mouse 1: DyLight 750 PEG Anti-CEA antibody conjugate, Mouse 2: Unconjugated DyLight 750 PEG, Mouse 3: DyLight 650 PEG Anti-CEA antibody conjugate, Mouse 4: Unconjugated DyLight 650 PEG. After 24 hours, the mice were sacrificed and a ventral vertical incision from the sternum to the lower abdomen was made to facilitate complete visceral exposure for whole body imaging. The mice were then imaged using the OV-100 Small Animal Imaging System (Olympus, Tokyo, Japan). In each instance images were taken with the liver in anatomical position and with the liver lifted up to expose the inferior surface of the liver.

### Animal Imaging

Mice were imaged using the Olympus OV100 Small Animal Imaging System (Olympus Corp. Tokyo, Japan), containing an MT-20 light source (Olympus Biosystems Planegg, Germany) and DP70 CCD camera (Olympus Corp. Tokyo, Japan). The OV100 was used due to its unique ability to accomplish high fidelity fluorescence imaging with variable magnification capabilities that allow for imaging of not only the whole animal but also at the subcellular level. The instrument incorporates a unique combination of variable magnification, high numerical aperture, and long working distance. Four individually optimized objective lenses, parcentered and parfocal provide a 10^5^ fold magnification range for seamless imaging of the entire body down to the subcellular level without disturbing the animal [23]. In addition, openings on the machine allow for delivery of inhalational anesthesia to the animal without allowing entry of outside light. This allows for capturing high quality in-vivo images with minimal morbidity to the animal by avoiding anesthetic injections. All images were analyzed using Image-J (National Institute of Health Bethesda, MD) and were processed with the use of Photoshop elements-11 (Adobe Systems Inc. San Jose, CA).

### Frozen Section Preparation

Mice were injected with 2.5 µmol (94 µg) anti-CEA antibody conjugated to PEGylated DyLight 650. 48 hours later, the antibody-labeled HT-29 RFP liver metastases were harvested. The tumor was placed in OCT compound and subsequently dipped into liquid nitrogen. Once the tumor was frozen, it was placed on a microtome and sliced into 5 µm sections and placed on a glass slide and immediately dipped in 95% ethanol for fixation. Alternate slides were stained with H&E for fluorescence and brightfield imaging. Fluorescence and confocal imaging were done with the FV1000 Confocal Laser Scanning Biological Microscope (Olympus Corp. Tokyo, Japan) [24].

### Statistical Analysis

All statistical analysis was done using SPSS software version 21 (IBM, Armonk, NY). For the pairwise comparisons within the 650 nm and 750 nm groups, quantitative variables were compared using the paired samples student t-test and confirmed with the Wilcoxon rank-sum test. A p-value <0.05 was considered significant. 95% confidence intervals obtained on analysis of the data were configured into all the error bars of the appropriate figures and graphs.

## Results

### Tissue biodistribution of PEGylated and non-PEGylated dyes conjugated to anti-CEA antibody

**Figure 2:**
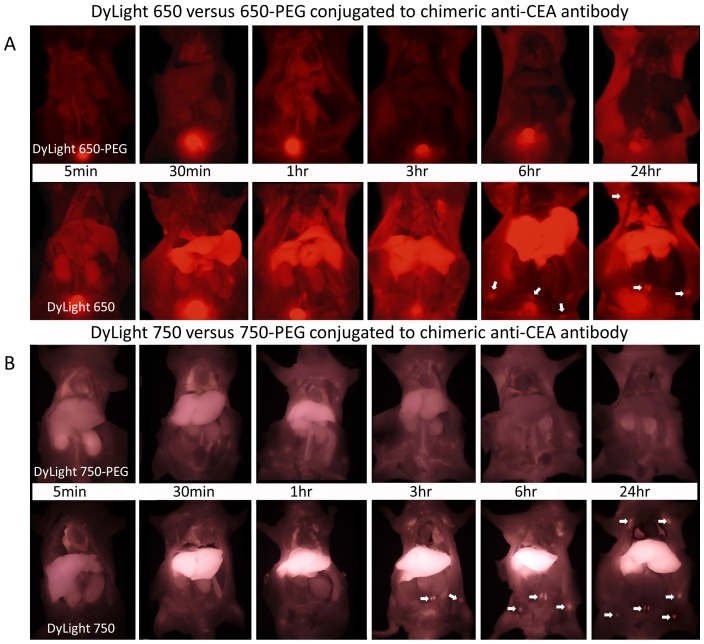
Whole body time sequence images of both PEGylated and non-PEGylated dye-anti-CEA antibody conjugates to evaluate total body biodistribution. After the mice are sacrificed at the indicated time-points, they were opened up from sternum to pubis to completely expose the viscera. In 2a, DyLight 650 anti-CEA antibody conjugate is evaluated, with images taken at different time-points. The top row is the PEGylated dye anti-CEA antibody conjugate, while the bottom row is the non-PEGylated anti-CEA antibody conjugate. With the PEGylated anti-CEA antibody conjugate, there is minimal to no accumulation of dye in the viscera, while in the non-PEGylated anti-CEA antibody conjugate, there is accumulation of dye in the reticulo-endothelial system organs such as the liver and lung. Arrows in the bottom row point to lymph nodes that retained and were labeled by the dye. In 2b, DyLight 750 is evaluated, with images taken at different time-points. The top row is the PEGylated dye anti-CEA antibody conjugate, while the bottom row is the non-PEGylated anti-CEA antibody conguate. With the PEGylated anti-CEA antibody conjugate, there was minimal to no accumulation of dye in the viscera, while in the non-PEGylated anti-CEA antibody conjugate there was accumulation of dye in the liver and lung. Arrows in the bottom row point to lymph nodes that retained and were labeled by the dye.

**Figure 3:**
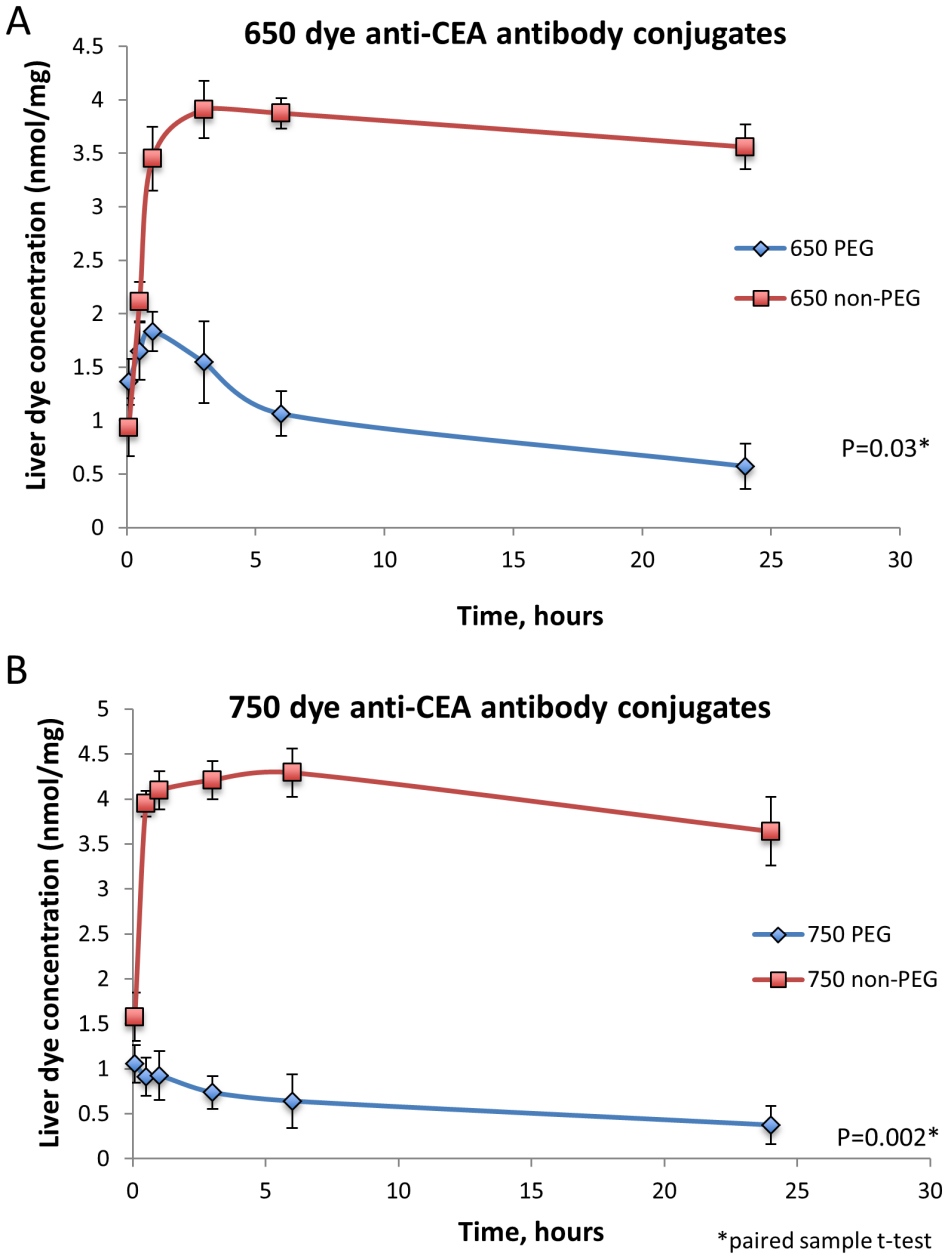
Liver tissue concentrations of the dye anti-CEA antibody conjugates over time. The liver was assayed to demonstrate tissue accumulation of the non-PEGylated and PEGylated 650 and 750 dyes that were conjugated to chimeric anti-CEA antibody. The non-PEGylated 650 (panel A) and 750 dye antibody conjugates (panel B) accumulated to significantly higher levels in the liver compared to their respective PEGylated dye antibody conjugates.

PEGylation significantly altered the biodistribution and elimination patterns of both the dyes. With PEGylation, little to no accumulation of dye conjugated to the antibody was noted in normal hepatic tissue (Figures 2 a&b). Dye was visualized in the kidneys, ureters and bladder indicating some component of renal elimination for both PEGylated and non-PEGylated dyes. Both PEGylated 650 and 750 dyes had significantly lower liver concentrations compared to the non-PEGylated counterparts in non-tumor-bearing mice with p = 0.03 for the 650 group and p = 0.002 for the 750 group (Figure 2 a&b and Figure 3).

### High-resolution labeling of liver metastases by PEGylated dyes conjugated to anti-CEA antibody

**Figure 4:**
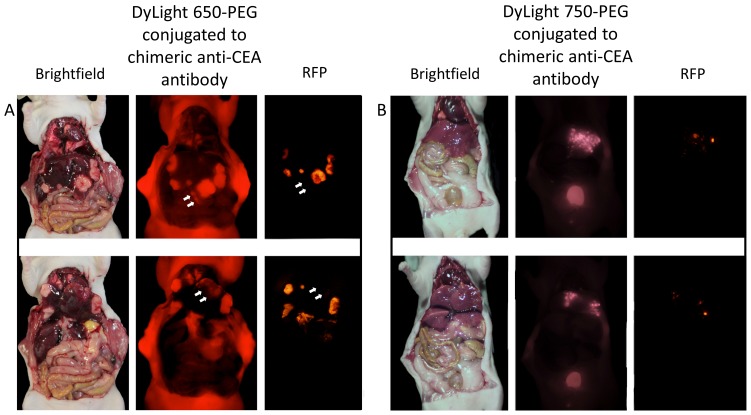
Liver metastasis models. In 4a, the PEGylated 650 anti-CEA antibody conjugate was injected into the mouse 24 hours prior to imaging. While the liver metastases are partially visible on bright field imaging, the anti-CEA antibody conjugated to PEGylated dye accurately and selectively labeled the tumors showing the true extent of disease. The RFP signal visualizes less of the tumor in comparison to the 650 nm signal from the anti-CEA chimeric antibody conjugated to PEGylated dyes, as demonstrated by the arrowheads. Any deep tumor or tumor overlain by liver tissue was not visualized with the RFP signal due to the poor penetration and hemoglobin quenching of the 550 nm signal. Upper and lower panels show same mouse with liver lifted up in lower panel in order to show additional view of liver metastases. In 4b, the PEGylated 750 anti-CEA antibody conjugate was injected into the mouse 24 hours prior to imaging. The liver metastases in this case are barely visible on bright field imaging. The PEGylated anti-CEA antibody conjugate however, brightly labels the metastases showing the true extent of disease. The RFP signal did not demonstrate the true extent of disease due to low tissue penetration and hemoglobin quenching when compared to the 750 nm signal. Upper and lower panels show same mouse with liver lifted up in lower panel in order to show additional view of liver metastases.

The non-PEGylated dyes accumulated extensively in the liver, which precluded imaging of metastatic disease to the liver. Therefore, we imaged mice bearing HT-29 colon cancer liver metastases using anti-CEA antibody labeled with 650-PEG (Figure 4a) or 750-PEG (Figure 4b). The liver metastases were readily visualized in the liver with minimal background fluorescence of normal liver (Figure 4 a & b). Furthermore, when labeled by the PEGylated dye-mAb complex, more of the metastatic tumor was visible when compared to the genetic RFP signal of the cancer cells (Figure 4 a & b).

### Comparison of labeling with PEGylated dyes conjugated to anti-CEA and histology to distinguish liver metastases

**Figure 5:**
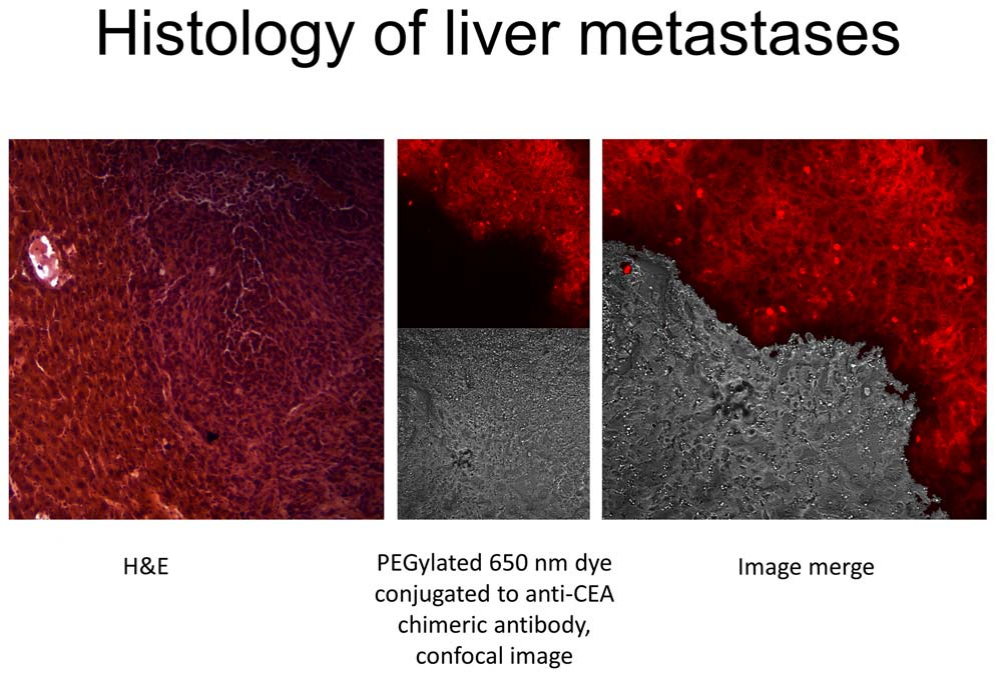
Demonstration of metastatic colon cancer cells adjacent to hepatic tissue. H&E, confocal and fluorescence images of adjacent tissue samples are shown (Confocal and fluorescence images from same slide). The tumor margin was much better delineated when employing fluorescence microscopy.

**Figure 6:**
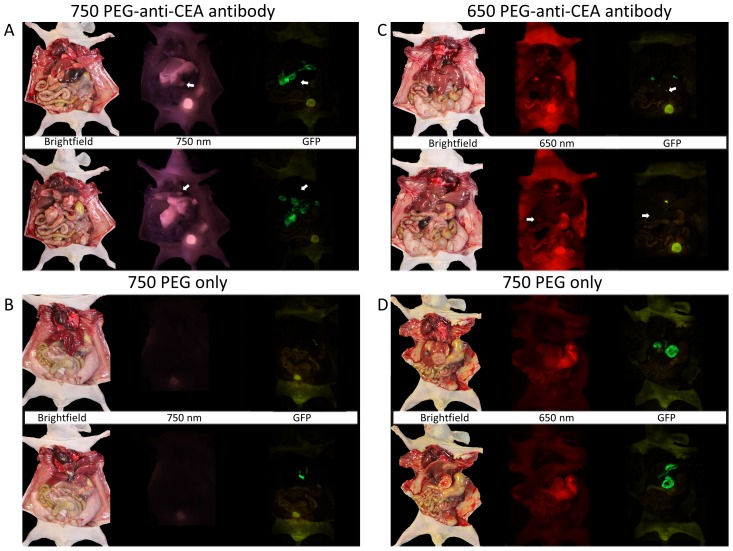
Specificity of anti-CEA antibody conjugated dyes as compared to free dyes. In all images from A to D, the top panel of images is taken with the liver in anatomical position and the bottom panel is taken with the liver flipped upward to enable visualization of the undersurface. Image A is taken 24-anti-CEA antibody conjugate. Imaging at 750 nm clearly delineates all the HT-29-GFP liver metastases which are not completely visible on imaging with brightfield or GFP imaging (see arrows). Image B is taken 24 hours after injection of the 750 PEG dye only. No visible tumor labeling is noted, with little to no visualization of the dye throughout the animal, despite the GFP signal of the tumor being clearly detected. Image C is taken 24 hours after injection of the 650 PEG-anti-CEA antibody conjugate. There is very precise and clear labeling of tumor that is detected at 650 nm, which correlates well with the GFP signal. With the liver in the anatomical position, the gallbladder is also noted emerging from the underside of the liver. On the underside of the liver, a tumor is detected that is not visible on either GFP or brightfield imaging (see arrows). Image D is taken 24 hours after injection of the 650PEG free dye. There is non-specific distribution of the dye noted at 650 nm, with no selectivity for the tumor despite there being a robust GFP signal.

The chimeric anti-CEA antibody conjugated with the PEGylated dyes labeled the liver metastases much more clearly than normal hepatic parenchyma and distinguished the metastases better than conventional H&E staining (Figure 5). The antibody-labeling was specific for the HT-29-GFP liver metastases since the corresponding free dyes did not show any tumor labeling (Figure 6).

## Discussion

In the present study, we demonstrated that PEG conjugation of NIR dyes conjugated to a chimeric anti-CEA antibody enhances imaging of liver metastases in nude-mouse models. With 4 mPEG chains conjugated to each dye molecule and 4–6 dye molecules conjugated to each mAb, this would have given each conjugated antibody molecule a minimum coverage of at least 16 mPEG chains. The change in biodistribution due to PEGylation of dyes conjugated to anti-CEA antibody results in less non-specific distribution of dye, allowing for a higher-signal-to-background ratio (SBR), especially in the reticuloendothelial organs compared to anti-CEA antibody conjugated to non-PEGylated dyes.

Regarding specificity, we have recently shown that the chimeric fluorophore-conjugated CEA antibody used in the present study demonstrated more effective labeling of human CEA-expressing cancer in tissue arrays and in patient-derived orthotopic xenograft (PDOX) nude models of human patient colon cancer. In this study, there was minimal labeling in the normal liver and lung tissue but significantly greater fluorescence signal intensity in labeled CEA-expressing colon, lung and pancreas tumor tissues. There was no labeling of CEA-negative tumors of the prostate, skin, ovary, uterus, brain, small intestine, kidney, stomach, and liver. There was also very minimal labeling of any tissue, normal or tumor, with the negative control IgG antibody [25].

In another experiment to demonstrate specificity of the anti-CEA antibody conjugated to PEGylated dyes, the free PEGylated dyes were compared to their anti-CEA-conjugated counterparts. There was a striking difference in that only the antibody-conjugated dyes labeled the liver metastases. The free PEGylated dyes demonstrated no labeling of the metastases. Thus the conjugated antibody with PEGylated dyes is highly specific to label the metastases.

The RFP label of the tumors was not as clearly discernible with less of the tumor being visualized when compared to both DyLight 650-PEG and 750-PEG labeling. Any deep tumors or tumors covered by liver tissue were unable to be visualized with the RFP signal.

Only two NIR dyes are currently FDA approved, ICG and methylene blue (MB). ICG fluoresces at ≈800 nm and MB, which when diluted to levels almost undetectable to the human eye fluoresces at ≈700 nm, a phenomenon known as ‘unquenching’ [26]–[28]. ICG permits imaging of intrahepatic and biliary structures, while MB permits imaging of both bile ducts and ureters [29], [30]. ICG has been most studied in liver imaging, but is non-specific [31], [32], contaminates the GI tract due to biliary secretion and is not very bright [33], and is unable to be covalently conjugated to targeting ligands [34].

The present study demonstrated that PEGylated NIR dyes conjugated to a chimeric tumor-specific antibody show promise in use for imaging of liver metastases with their high SBR within the liver, the ability to bind to a target ligand and lack of GI contamination due to absence of biliary excretion. The dyes show promise in imaging of metastatic or intrinsic hepatobiliary malignancies and should be useful for imaging of tumors in any tissue of interest as the dyes minimally accumulate non-specifically and they are very bright with high SBR and should have great potential for fluorescence-guided surgery.

## References

[1] Hoffman RM (2005) The multiple uses of fluorescent proteins to visualize cancer in vivo. Nat Rev Cancer 5: 796–806. 16195751 10.1038/nrc1717

[2] Bouvet M, Hoffman RM (2011) Glowing tumors make for better detection and resection. Sci Transl Med 3: 110fs110. 10.1126/scitranslmed.3003375 22116932

[3] Lee BT, Hutteman M, Gioux S, Stockdale A, Lin SJ, et al (2010) The FLARE intraoperative near-infrared fluorescence imaging system: a first-in-human clinical trial in perforator flap breast reconstruction. Plast Reconstr Surg 126: 1472–1481. 21042103 10.1097/PRS.0b013e3181f059c7 PMC2974179

[4] Okuda T, Yoshioka H, Kato A (2012) Fluorescence-guided surgery for glioblastoma multiforme using high-dose fluorescein sodium with excitation and barrier filters. J Clin Neurosci 19: 1719–1722. 23036170 10.1016/j.jocn.2011.12.034

[5] Spinoglio G, Priora F, Bianchi PP, Lucido FS, Licciardello A, et al (2013) Real-time near-infrared (NIR) fluorescent cholangiography in single-site robotic cholecystectomy (SSRC): a single-institutional prospective study. Surg Endosc 27: 2156–2162. 23271272 10.1007/s00464-012-2733-2

[6] van Dam GM, Themelis G, Crane LM, Harlaar NJ, Pleijhuis RG, et al (2011) Intraoperative tumor-specific fluorescence imaging in ovarian cancer by folate receptor-alpha targeting: first in-human results. Nat Med 17: 1315–1319. 21926976 10.1038/nm.2472

[7] Verbeek FP, van der Vorst JR, Schaafsma BE, Swijnenburg RJ, Gaarenstroom KN, et al.. (2013) Intraoperative Near Infrared Fluorescence Guided Identification of the Ureters Using Low Dose Methylene Blue: A First in Human Experience. J Urol. 10.1016/j.juro.2013.02.3187 PMC3686854 23466242

[8] Metildi CA, Kaushal S, Hardamon CR, Snyder CS, Pu M, et al (2012) Fluorescence-guided surgery allows for more complete resection of pancreatic cancer, resulting in longer disease-free survival compared with standard surgery in orthotopic mouse models. J Am Coll Surg 215: 126–135 discussion 135–126. 22632917 10.1016/j.jamcollsurg.2012.02.021 PMC3383387

[9] Metildi CA, Hoffman RM, Bouvet M (2013) Fluorescence-guided surgery and fluorescence laparoscopy for gastrointestinal cancers in clinically-relevant mouse models. Gastroenterol Res Pract 2013: 290634. 23533387 10.1155/2013/290634 PMC3590746

[10] Metildi CA, Kaushal S, Snyder CS, Hoffman RM, Bouvet M (2013) Fluorescence-guided surgery of human colon cancer increases complete resection resulting in cures in an orthotopic nude mouse model. J Surg Res 179: 87–93. 23079571 10.1016/j.jss.2012.08.052 PMC3518733

[11] Kishimoto H, Zhao M, Hayashi K, Urata Y, Tanaka N, et al (2009) In vivo internal tumor illumination by telomerase-dependent adenoviral GFP for precise surgical navigation. Proc Natl Acad Sci U S A 106: 14514–14517. 19706537 10.1073/pnas.0906388106 PMC2732810

[12] Kaushal S, McElroy MK, Luiken GA, Talamini MA, Moossa AR, et al (2008) Fluorophore-conjugated anti-CEA antibody for the intraoperative imaging of pancreatic and colorectal cancer. J Gastrointest Surg 12: 1938–1950. 18665430 10.1007/s11605-008-0581-0 PMC4396596

[13] McElroy M, Kaushal S, Luiken GA, Talamini MA, Moossa AR, et al (2008) Imaging of primary and metastatic pancreatic cancer using a fluorophore-conjugated anti-CA19-9 antibody for surgical navigation. World J Surg 32: 1057–1066. 18264829 10.1007/s00268-007-9452-1 PMC4378829

[14] Metildi CA, Kaushal S, Lee C, Hardamon CR, Snyder CS, et al. (2012) An LED light source and novel fluorophore combinations improve fluorescence laparoscopic detection of metastatic pancreatic cancer in orthotopic mouse models. J Am Coll Surg 214: : 997–1007 e1002. 10.1016/j.jamcollsurg.2012.02.009 PMC3360832 22542065

[15] Tran Cao HS, Kaushal S, Metildi CA, Menen RS, Lee C, et al (2012) Tumor-specific fluorescence antibody imaging enables accurate staging laparoscopy in an orthotopic model of pancreatic cancer. Hepatogastroenterology 59: 1994–1999. 22369743 10.5754/hge11836 PMC4096574

[16] Maawy AA, Hiroshima Y, Kaushal S, Luiken GA, Hoffman RM, et al (2013) Comparison of a chimeric anti-carcinoembryonic antigen antibody conjugated with visible or near-infrared fluorescent dyes for imaging pancreatic cancer in orthotopic nude mouse models. J Biomed Opt 18: 126016. 24356647 10.1117/1.JBO.18.12.126016 PMC3868446

[17] Veronese FM, Morpurgo M (1999) Bioconjugation in pharmaceutical chemistry. Farmaco 54: 497–516. 10510847 10.1016/s0014-827x(99)00066-x

[18] Bailon P, Won CY (2009) PEG-modified biopharmaceuticals. Expert Opin Drug Deliv 6: 1–16. 19236204 10.1517/17425240802650568

[19] Delgado C, Francis GE, Fisher D (1992) The uses and properties of PEG-linked proteins. Crit Rev Ther Drug Carrier Syst 9: 249–304. 1458545

[20] Harris JM, Martin NE, Modi M (2001) Pegylation: a novel process for modifying pharmacokinetics. Clin Pharmacokinet 40: 539–551. 11510630 10.2165/00003088-200140070-00005

[21] Katz MH, Takimoto S, Spivack D, Moossa AR, Hoffman RM, et al (2003) A novel red fluorescent protein orthotopic pancreatic cancer model for the preclinical evaluation of chemotherapeutics. J Surg Res 113: 151–160. 12943825 10.1016/s0022-4804(03)00234-8

[22] Bouvet M, Tsuji K, Yang M, Jiang P, Moossa AR, et al (2006) In vivo color-coded imaging of the interaction of colon cancer cells and splenocytes in the formation of liver metastases. Cancer Res 66: 11293–11297. 17145875 10.1158/0008-5472.CAN-06-2662

[23] Yamauchi K, Yang M, Jiang P, Xu M, Yamamoto N, et al (2006) Development of real-time subcellular dynamic multicolor imaging of cancer-cell trafficking in live mice with a variable-magnification whole-mouse imaging system. Cancer Res 66: 4208–4214. 16618743 10.1158/0008-5472.CAN-05-3927

[24] Uchugonova A, Zhao M, Weinigel M, Zhang Y, Bouvet M, et al (2013) Multiphoton tomography visualizes collagen fibers in the tumor microenvironment that maintain cancer-cell anchorage and shape. J Cell Biochem 114: 99–102. 22886742 10.1002/jcb.24305

[25] Metildi CA, Kaushal S, Luiken GA, Talamini MA, Hoffman RM, et al (2014) Fluorescently labeled chimeric anti-CEA antibody improves detection and resection of human colon cancer in a patient-derived orthotopic xenograft (PDOX) nude mouse model. J Surg Oncol 109: 451–458. 24249594 10.1002/jso.23507 PMC3962702

[26] Gioux S, Choi HS, Frangioni JV (2010) Image-guided surgery using invisible near-infrared light: fundamentals of clinical translation. Mol Imaging 9: 237–255. 20868625 PMC3105445

[27] Tanaka E, Chen FY, Flaumenhaft R, Graham GJ, Laurence RG, et al (2009) Real-time assessment of cardiac perfusion, coronary angiography, and acute intravascular thrombi using dual-channel near-infrared fluorescence imaging. J Thorac Cardiovasc Surg 138: 133–140. 19577070 10.1016/j.jtcvs.2008.09.082 PMC2706783

[28] Verbeek FP, van der VorstJR, Schaafsma BE, Hutteman M, Bonsing BA, et al (2012) Image-guided hepatopancreatobiliary surgery using near-infrared fluorescent light. J Hepatobiliary Pancreat Sci 19: 626–637. 22790312 10.1007/s00534-012-0534-6 PMC3501168

[29] Matsui A, Tanaka E, Choi HS, Kianzad V, Gioux S, et al (2010) Real-time, near-infrared, fluorescence-guided identification of the ureters using methylene blue. Surgery 148: 78–86. 20117811 10.1016/j.surg.2009.12.003 PMC2886170

[30] Matsui A, Tanaka E, Choi HS, Winer JH, Kianzad V, et al (2010) Real-time intra-operative near-infrared fluorescence identification of the extrahepatic bile ducts using clinically available contrast agents. Surgery 148: 87–95. 20117813 10.1016/j.surg.2009.12.004 PMC2886157

[31] Achilefu S (2010) The insatiable quest for near-infrared fluorescent probes for molecular imaging. Angew Chem Int Ed Engl 49: 9816–9818. 21089086 10.1002/anie.201005684 PMC3046463

[32] Lee H, Mason JC, Achilefu S (2006) Heptamethine cyanine dyes with a robust C-C bond at the central position of the chromophore. J Org Chem 71: 7862–7865. 16995699 10.1021/jo061284u

[33] Ohnishi S, Lomnes SJ, Laurence RG, Gogbashian A, Mariani G, et al (2005) Organic alternatives to quantum dots for intraoperative near-infrared fluorescent sentinel lymph node mapping. Mol Imaging 4: 172–181. 16194449 10.1162/15353500200505127

[34] Kobayashi H, Ogawa M, Alford R, Choyke PL, Urano Y (2010) New strategies for fluorescent probe design in medical diagnostic imaging. Chem Rev 110: 2620–2640. 20000749 10.1021/cr900263j PMC3241938

